# Malaria Elimination―Not Just a Bunch of Hocus-Pocus

**DOI:** 10.3201/eid2502.AC2502

**Published:** 2019-02

**Authors:** Byron Breedlove, Paul M. Arguin

**Affiliations:** Centers for Disease Control and Prevention, Atlanta, Georgia, USA

**Keywords:** art science connection, emerging infectious diseases, art and medicine, about the cover, Quintus Serenus Sammonicus, Liber Medicinalis, public health, malaria, parasites, malaria elimination―not just a bunch of hocus-pocus, protozoa, mosquito-borne diseases, vector-borne diseases, zoonotic infections, public health, World Health Organization

**Figure:**
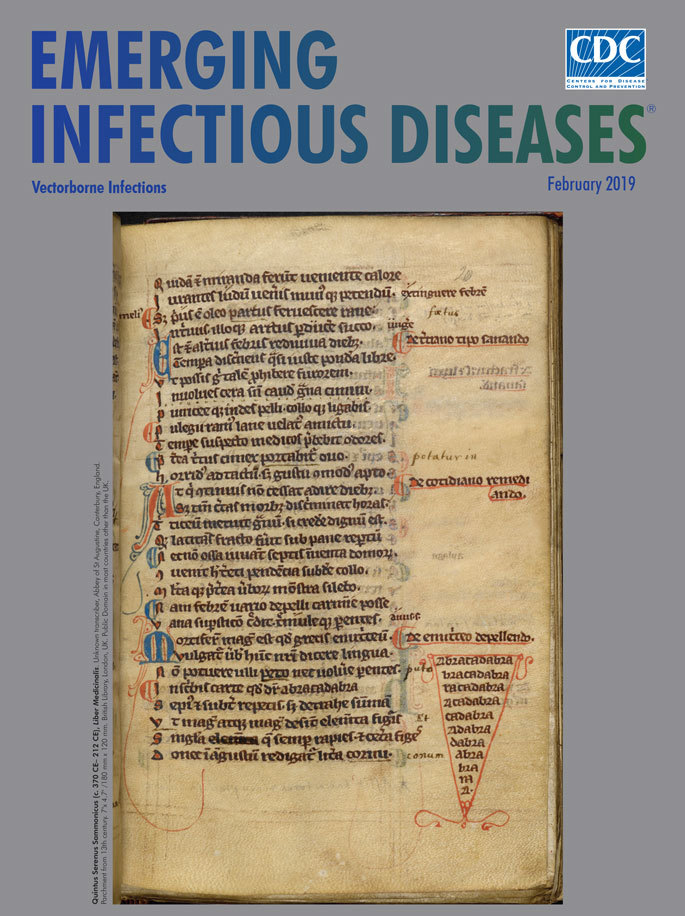
**Quintus Serenus Sammonicus (c. 370 CE–212 CE), *Liber Medicinalis*. **Unknown transcriber, Abbey of St. Augustine, Canterbury, England. Parchment from 13th century. 7 in × 4.7 in/180 mm × 120 mm. British Library, London, UK. Public Domain in most countries other than the United Kingdom.

*Abracadabra*! This ubiquitous incantation remains a staple of stage magicians, children’s stories, and purveyors of pseudoscience. The exact origin of the term engenders debate, and pundits have suggested various ancient Aramaic, Hebrew, and Latin terms as the source. What is known is that its first appearance in print is found in the surviving fragments of the third century CE book *Liber Medicinalis* (sometimes known as *De Medicina Praecepta Saluberrima*) written by Quintus Serenus Sammonicus. Though Serenus was the physician to the Roman Emperor Caracalla and considered “the learned man of his age,” few details of his life are known.^1^

Following the practice of his time, Serenus composed his teachings as didactic poetry. The surviving fragment of *Liber Medicinalis* includes popular treatments, remedies, and antidotes written in verse.

Among those remedies, Serenus proposed a magical procedure based on the wizardly word *Abracadabra* for treating “semitertian” fever, known today as malaria. That malady devastated ancient Rome and was sometimes also called “rage of the Dog Star” as the ascendance of Sirius presaged the oppressive heat and humidity thought to cause fever and illness. Some wealthy Romans sought to escape this scourge by moving to villas they had built in the hills away from the “bad air” (malum aeris in Latin) emanating from the marshes and wetlands surrounding Rome. With a bit of alchemical panache, Serenus offered another approach documented in chapter 51 of the *Liber Medicinalis:*

*Inscribis chartae, quod dicitur Abracadabra:*

*Saepius et subter repetas, sed detrahe summae,*

*Et magis atque magis desint elementa figuris:*

*Singula quae semper rapies et coetera figes, *

*Donec in angustam redigatur litera conum. *

*His lino nexis collum redimire memento*

Various translations of the Latin are available. This one comes from by A. C. Wootton, who, for three decades, served as editor of the trade journal *Chemist and Druggist*.

“Write several times on a piece of paper the word ‘Abracadabra,’ and repeat the words in the lines below but take away letters from the complete word and let the letters fall away one at a time in each succeeding line. Take these away ever, but keep the rest until the writing is reduced to a narrow cone. Remember to tie these papers with flax and bind them round the neck.” 

The idea underscoring this magical thinking was that by making the letters disappear, the illness would likewise vanish. This month’s cover image shows a 13th century transcription of this page from *Liber Medicinalis* and comes from the Benedictine Abbey of St. Augustine, Canterbury, England. Like a slice of pie resting in the margin of the book, the *ABRACADABRA* cone is visible near the lower right of the parchment. The original Latin, rendered painstakingly in ornate Gothic and Gothic cursive, explains Serenus’ process for creating the triangular charm inscribed with the enchantment and for wearing it as an amulet. Perhaps to hedge his bets, Serenus also suggested smearing lion’s fat on one’s body or wearing a domestic cat’s skin festooned with jewels to ward off these fevers.

Feline byproducts, bejeweled or otherwise, magic words, and amulets all failed, and malaria continues to be one of the most severe global public health problems. Fortunately, though, the scientists of today have been a bit more effective through core interventions of surveillance, diagnosis, prevention, and treatment. In many countries, using artemisinin-based combination therapy, undertaking vector control measures such as using long-lasting insecticides on bed nets and interior walls of houses, and strengthening public health infrastructure have successfully reduced cases and deaths.

While progress is starting to plateau in many highly malaria-endemic countries, several other countries either have eliminated, or are on the verge of eliminating, malaria. Paraguay and Uzbekistan recently celebrated their initiation into the malaria elimination club, receiving certification by the World Health Organization (WHO). El Salvador and China have recently reached zero cases. Both countries will receive their official WHO certification when they have “proven, beyond reasonable doubt, that the chain of local transmission of all human malaria parasites has been interrupted nationwide for at least the past 3 consecutive years; and that a fully functional surveillance and response system that can prevent re-establishment of indigenous transmission is in place.”

In just the past 10 years, the number of malaria-endemic countries has decreased from 108 (in 2008) to 90 (in 2018). In 2017, about half of all of the remaining malaria-endemic countries had reported fewer than 10,000 cases per year. WHO has outlined a strategy to continue paring down that list of malaria-endemic countries, one by one, with ambitious targets through 2030. Peering into our crystal ball, we hope, one day, to see that final country on the list like Serenus’ ultimate letter A and then *Abracadabra* ―all gone.
